# Sprengel deformity: What is the functional outcome of conservative treatment versus surgical correction?

**DOI:** 10.1186/s13023-025-03544-3

**Published:** 2025-01-22

**Authors:** C. Antfang, A. Frommer, G. Gosheger, G. Toporowski, A. Laufer, J. D. Rölfing, R. Roedl, B. Vogt

**Affiliations:** 1 Paediatric Orthopaedics, Deformity Reconstruction and Foot Surgery, General Orthopaedics and Tumour Orthopaedics, Muenster University Hospital, Albert-Schweitzer-Campus 1, Muenster, 48149 Germany; 2 General Orthopaedics and Tumour Orthopaedics, Muenster University Hospital, Muenster, Germany; 3 Children’s Orthopaedics and Reconstruction, Aarhus University Hospital, Aarhus, Denmark

**Keywords:** Sprengel deformity, Surgery, Green procedure, Scapula, Shoulder, Children, Adolescents, Cavendish, Rigault, EQ-5D-Y, QuickDASH

## Abstract

**Background:**

Sprengel deformity is a rare congenital malformation of the scapula defined by malposition during embryonic development. Affected individuals have limited range of motion of the shoulder and torticollis. Surgical reconstruction is an option to treat patients with severe deformity and functional impairment. This retrospective single centre study evaluated 19 patients with 21 Sprengel deformities treated from 2016 to 2023. 11/19 patients had mild ROM limitations of the affected shoulder with a median abduction of 130° (interquartile range (IQR) 100–150) and were treated conservatively. 8/19 patients with severe Sprengel deformity and functional impairment underwent surgery (median age 6 years (IQR 4–6)). Surgery was conducted in a modified technique according to Green. The Cavendish and Rigault scores were employed to analyse function, cosmesis and the radiographic location of the scapula. Patient reported outcome measurements (EQ-5D-Y and the quick Disabilities of the Arm, Shoulder and Hand Questionnaire) were both administered at the latest follow-up.

**Results:**

Patients treated conservatively had a median abduction of the affected arm of 130° (IQR 100–150) and a median Cavendish and Rigault score of both 2 (IQR 2–3 and 1.3-2, respectively). In the surgery group the median abduction improved by 45° (IQR 28–53) from 90° (IQR 90–90) preoperatively to 135° (IQR 120–140) 3 months postoperatively and was 110° (IQR 108–128) at latest follow-up. The median Cavendish score improved from 4 (IQR 2–4) to 1 (IQR 1–2). The median Rigault score was lowered from 3 (IQR 3–3) to 1 (IQR 1–2). The median time to return to daily life was 3 months (IQR 2.2–3.5). The median quickDASH score was 11.4 (IQR 7–31) in the surgical cohort and 9.1 (IQR 5–22) in the conservative cohort at median maximum follow-up of 62 months (IQR 22–118). The median EQ-VAS (Visual Analogue Scale) score was 81/100 (IQR 79–85/100) in the surgical cohort and 80/100 (IQR 59–95/100) in the non-surgical cohort. 4/8 patients treated surgically had fully reversible complications.

**Conclusions:**

Surgical treatment of severe Sprengel deformity improves abduction of the affected shoulder and reduces disability in daily life. Patients with mild Sprengel deformity can have very good function of the shoulder and should not be considered for surgery.

## Background

Sprengel deformity is a rare congenital malformation of the scapula, which was first described in 1863 by Eulenberg [1] and later by Sprengel in a small case series [2]. It is defined by a malplacement of the scapula during embryonic development. While most appendicular bones derive only from one tissue layer, the scapula derives from both the dermomyotome, somatopleure, and neural crest and thus adding to the pathogenetic complexity of the malformation. Once formed, it descends from its origin at the brachio-thoracic-axial level to a more caudally position at the posterior thorax [3–6]. In affected individuals the scapula is elevated and medialised with outward rotation resulting in limited shoulder abduction. The scapula is smaller, and its medial border is shortened with contractures of the attached muscles, especially the levator scapulae and the rhomboids [7, 8]. In one third to half of affected individuals an additional omovertebral connection can be found, which usually presents as a true bone or a fibrous tissue link between the scapula and the cervical spine [8–10].

The deformity often remains undetected after birth and is more commonly diagnosed in toddlers. Affected individuals present with torticollis and elevated shoulder impairing function and cosmetic appearance. Especially abduction and elevation of the affected shoulder are limited to less than 90° in many patients [8, 11, 12]. Only severe deformities with limitations in range of motion of the affected shoulder that impair daily activity should be considered for surgery [8].

Next to the operation technique first described by Woodward, surgery according to Green is one of the most employed techniques to correct Sprengel deformity [13, 14]. The technique according to Green uses a midline incision and a muscle release at the scapular insertion. A possibly present omovertebral bone needs to be resected first before the scapula can be lowered into a soft tissue pocket made of the latissimus dorsi. It is kept in position by a transcostal fixation while the muscles are reinserted in a more cranial position [15]. A prophylactic clavicle osteotomy to prevent nerve lesions of the brachial plexus is optional, yet controversially discussed [8].

This study aims to retrospectively evaluate the functional, cosmetic, and radiographic outcome as well as quality of life of patients who either underwent non-operative or operative treatment of Sprengel deformity.

## Methods

### Study setting, patients, and indications

This is a retrospective single centre study of 19 patients (10 female, 9 male) with 21 Sprengel deformities presenting at a tertiary referral university hospital from 2016 to 2022. Median age at first consultation was 5 years (interquartile range (IQR) 2–7) in the entire cohort and 3 years (IQR 1.5–6.7) in the surgical sub-cohort. The median age at surgery was 6 years (IQR 4–6). The median follow-up of the study was 62 months (IQR 36–114). Three patients presented themselves only once in our institution since they showed only mild forms and surgical correction was therefore not considered. One patient is scheduled for surgery.

Of all 19 patients only three had no associated congenital anomaly. With seven patients each, Klippel-Feil syndrome and scoliosis were the two most common musculoskeletal anomalies. An omovertebral bone was present in five patients. Further associations included malformations of the renal tract and spine malformations. A detailed overview is presented in Table 1.

**Table 1:** Characteristics of the 21 patients with varying severities of Sprengel deformity

Patient	Age (years) at time of first presentation or surgery	Sex (F = female, M = male)	Side (*R* = right, L = left)	Cavendish/ Rigault pre-operative	Cavendish/ Rigault post-operative	Shoulder abduction in ° pre-operative	Shoulder abduction in ° post-operative	Surgery time in minutes	Complications	Associated Anomalies	Follow-up (months)	Equation 5D-Y state	Equation 5D-Y VAS
1	7	F	R	2 / 2	-	160	-	-		Block vertebra, Scoliosis, Omovertebral bone	143	11123	48
2	10	F	L	2 / 1	-	130	-	-		Scoliosis	128	11222	50
3	7	F	L	2 / 2	-	90	-	-		Scoliosis	98	11122	67
4	8	M	R	3 / 2	-	150	-	-		Klippel-Feil syndrome	49	11121	80
5	3	F	L	1 / 1	-	150	-	-		None	49		
6	7	M	R	2 / 1	-	90	-	-		Scoliosis	0		
7	2	M	R	1 / 1	-	90	-	-		None	0		
8	5	F	R	2 / 2	-	170	-	-		None	16	11111	100
9	3	M	L	3 / ?	-	110	-	-		Half vertebra 1st thoracic vertebra	3	12111	90
10	7	M	L	1 / ?	-	130	-	-		None	23	11113	100
11*	5	F	L R	3 / 2 4 / 3	- -	100 100	-	-		Klippel-Feil syndrome	Lost to Follow-up		
12 (a)	4	M	R	3 / 2	-	130	-	-		Klippel-Feil syndrome;	111		
(b)			L	4 / 3	2 / 2	90	120	107	Radial palsy (reversible)	Omovertebral bone		11222	83
13	6	F	R	4 / 3	1 / 1	90	140	n/a	-	Klippel-Feil syndrome; Omovertebral bone	137	12222	80
14	6	M	R	4 / 3	1 / 2	60	140	93	-	Klippel-Feil syndrome; Scoliosis	118	11121	95
15	3	F	L	4 / 3	2 / 2	100	140	86	-	Nephrological anomaly; Omovertebral bone	85		
16	4	M	R	2 / 2	1 / 1	90	150	64	Pneumo-thorax	Klippel-Feil syndrome	73	22222	40
17	5	F	L	3 / 3	1 / 1	90	130	96	Seroma	Malformations upper spine and ribs; Omovertebral bone	71	11111	85
18	16	M	R	3 / 3	3 / 3	90	90	88	Brachial palsy (reversible)	Scoliosis	34	11221	78
19	16	F	R	4 / 3	1 / 1	90	110	97	-	Klippel-Feil syndrome; Omovertebral bone	38		

Pat. 12 (b) to 19 received surgery. Pat. 12 had bilateral Sprengel deformity but received surgery only on the left shoulder. *Pat. 11 is scheduled for surgery. n/a = not available

In general, at first consultation patients and families were counselled against surgery when the affected individual was adolescent and/or with minor functional impairment of the shoulder during daily activity. Surgery was regarded as a feasible treatment for patients younger than 8 years with functional and/or cosmetic impairment during daily activity. Since correction of Sprengel deformity is elective surgery, the final decision was left to the families which led to the inclusion of two 16-year-old patients in the surgery group.

**Fig. 1:**
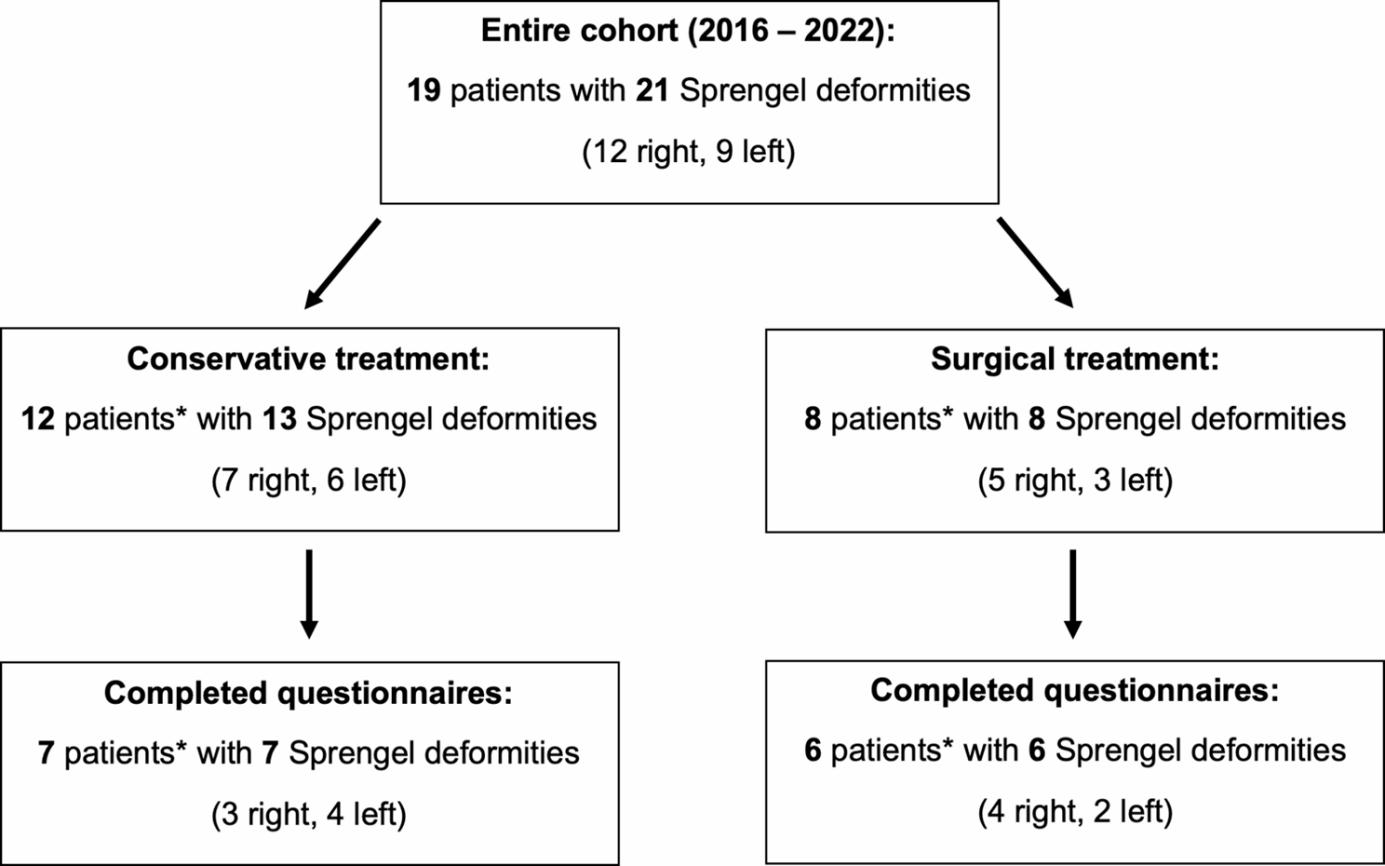
Reporting of Observational studies in Epidemiology (STROBE) diagram detailing the inclusion and exclusion criteria for the study cohort. *In one of the two patients with bilateral Sprengel deformity, the right side was treated conservatively and the left side surgically

The study findings are reported according to the *Strengthening the Reporting of Observational Studies in Epidemiology* (STROBE) guidelines [16] (Fig. 1).

### Pre- and postoperative assessment

#### Clinical assessment

The clinical outcome was evaluated by measuring the active and passive range of motion of the affected shoulder at first consultation and at maximum follow-up. The cosmetic appearance of the deformity was subclassified clinically according to Cavendish [17] (Table 2).

**Table 2:** Classification of sprengel deformity according to cavendish and rigault

**Cavendish**	**1**	Shoulders are level. Deformity is not visible when the patient is dressed.
	**2**	Shoulders are almost level. Deformity is visible as a lump in the web of the neck when the patient is dressed.
	**3**	Shoulder is elevated 2–5 cm. Deformity is easily seen.
	**4**	Shoulder is elevated. The superior angle of the scapula lies near the occiput.
**Rigault**	**1**	Superomedial angle lower than T2 but above T4 transverse process.
	**2**	Superomedial angle located between C5 and T2 transverse process.
	**3**	Superomedial angle above C5 transverse process.

#### Patient reported outcome measurement

At maximum follow-up all patients were asked to complete the short version of the Disabilities of the Arm, Shoulder and Hand Questionnaire (quickDASH) and the 5-level EQ-5D version (EQ-5D-Y) introduced by the EuroQol Group. The quickDASH as an adaptation of the DASH is the best-tested and widest spread instrument to assess shoulder function in a region-specific manner [18]. It ranges from 0 to 100 with lower values indicating good to excellent function and 100 meaning total loss of function.

The EQ-5D questionnaire consists of 2 pages: the EQ-5D descriptive system and the EQ visual analogue scale (EQ VAS). Mobility, self-care, usual activities, pain/discomfort, and anxiety/depression are part of the EQ-5D descriptive system. Each modality has five dimensions linked to the digits 1–5 [19].

#### Radiographic assessment

**Fig. 2:**
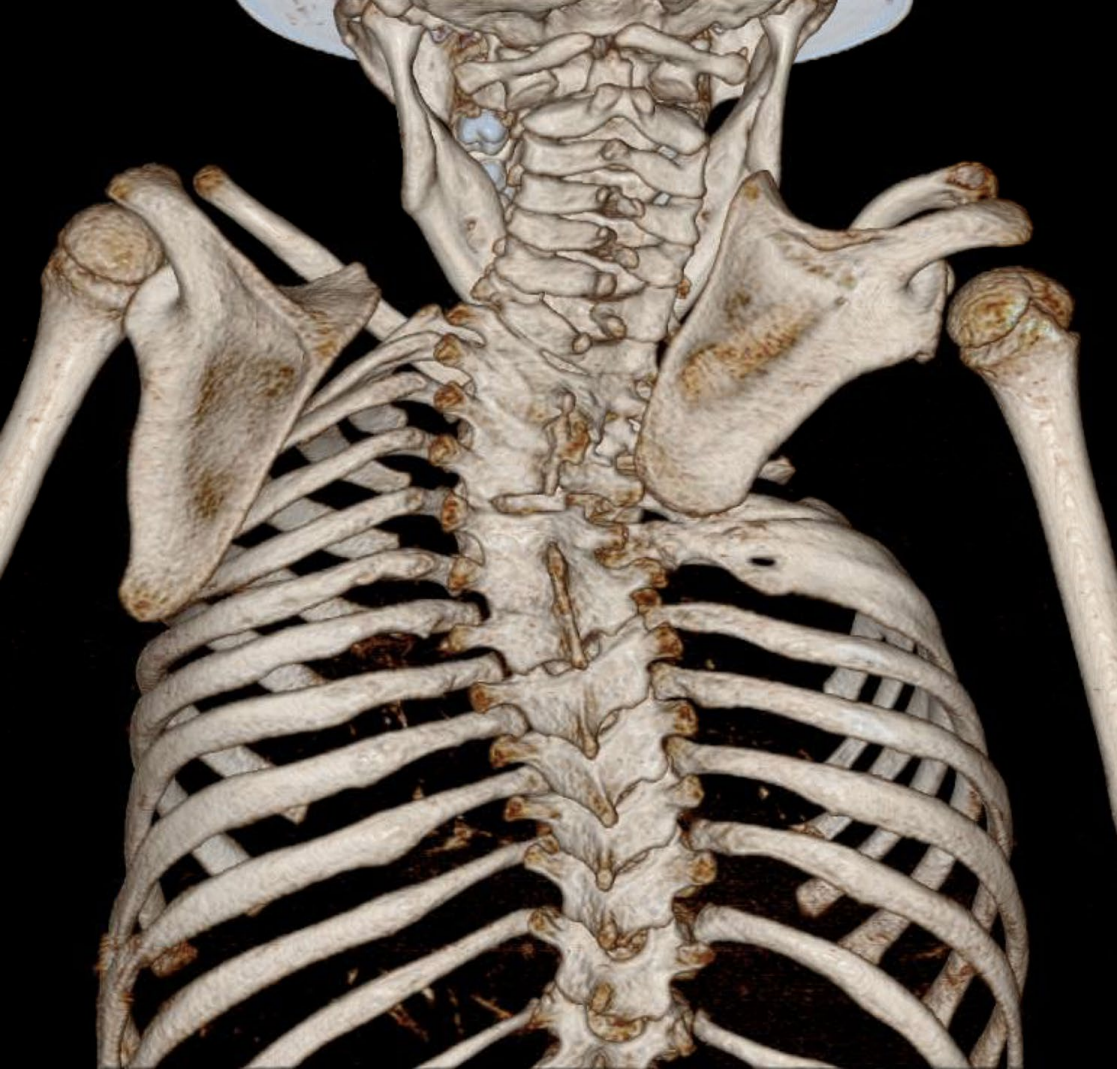
3D-CT scan of a child with unilateral Sprengel deformity of the right shoulder. Preoperative imaging showing Rigault score 3 and associated congenital scoliosis and rib anomalies on the right side

The shape and position of the scapula and shoulder was retrospectively and descriptively analysed on preoperative radiographs (anteroposterior and Y-view). Severity of the deformity was graded according to Rigault dependent on the position of the scapula in relation to the cervical vertebrae [20] (Table 2). For further planning and to identify additional malformations all surgically treated patients received a preoperative three-dimensional computer tomography (3D-CT) scan of the shoulder girdle and the thoracic aperture (Fig. 2).

### Surgical technique, perioperative management, and follow-up

Surgery was conducted on five right and three left shoulders and all operations were carried out by the same surgeon (RR). Whenever present an omovertebral bone was resected in the respective patients (Pat. 12b, 13, 15, 17, 19). The Green procedure was performed in a modified technique on all eight patients. This technique was chosen by the surgeon since it allows the maximum derotation of the scapula and is therefore presumably the most effective [21]. One 300 ml concentrate of red blood cells was provided by the institutional blood bank as a backup for each operation. Placed in a prone position under general anaesthesia a long median incision across the back was conducted. Following the Green procedure the trapezoid and rhomboid muscles were released close to the scapula. If present, an omovertebral bone or fibrous band at the superomedial angle of the scapula was resected. In accordance with the modified technique the scapula was then lowered and derotated into a pocket formed with the latissimus dorsi instead of using a percutaneous traction system. To fix the scapula in the corrected position, the inferior angle got connected to the 8th or 9th rib using a strong suture made of ultra-high-molecular-weight polyethylene (FiberWire^®^, Arthrex, Naples, FL, USA). Finally, the released muscles were reattached more cranially at the scapula to keep the new position.

In this series the soft tissue tension after correction was intraoperatively assessed and estimated as adequate so that a prophylactic osteotomy of the clavicle was not routinely conducted. Following surgery, the arm of the affected shoulder was immobilised in a sling for 7 to 10 days, after which pain adapted free mobility of the shoulder and physiotherapy was initiated.

### Statistical report

SPSS Statistics v28.0.1.0 (IBM, Chicago, IL, USA) was used for all statistical analyses. As the assessment of the data distribution pattern with the Shapiro-Wilk test revealed that all continuous variables were non-parametric, descriptive analysis was performed using median with IQR presented as 25th -75th percentile. Absolute numbers with percentages were given for binary variables.

## Results

### Clinical outcome

Of the 21 Sprengel deformities in 19 patients, ten patients were treated conservatively (e.g. physiotherapy and ergotherapy if necessary), eight underwent surgery and one is scheduled for surgery. At first consultation the median abduction of the affected shoulder in the non-operative cohort (*n* = 11) was 130° (IQR 100–150), while the median preoperative abduction in the surgical cohort was 90° (IQR 90–90).

At maximum follow-up the median abduction of the affected shoulder in the non-operative cohort was still 130° (IQR 97–152). In the surgical cohort the median abduction increased by median 45° (IQR 28–53) to a median of 135° (IQR 120–140) 3 months postoperatively but re-decreased to median 110° (IQR 107–120) at maximum follow-up. The two oldest patients (16 years; Pat. 18 and 19; Table 1) both showed the least improvement of shoulder abduction after surgery (0° and 20°, respectively).

**Fig. 3:**
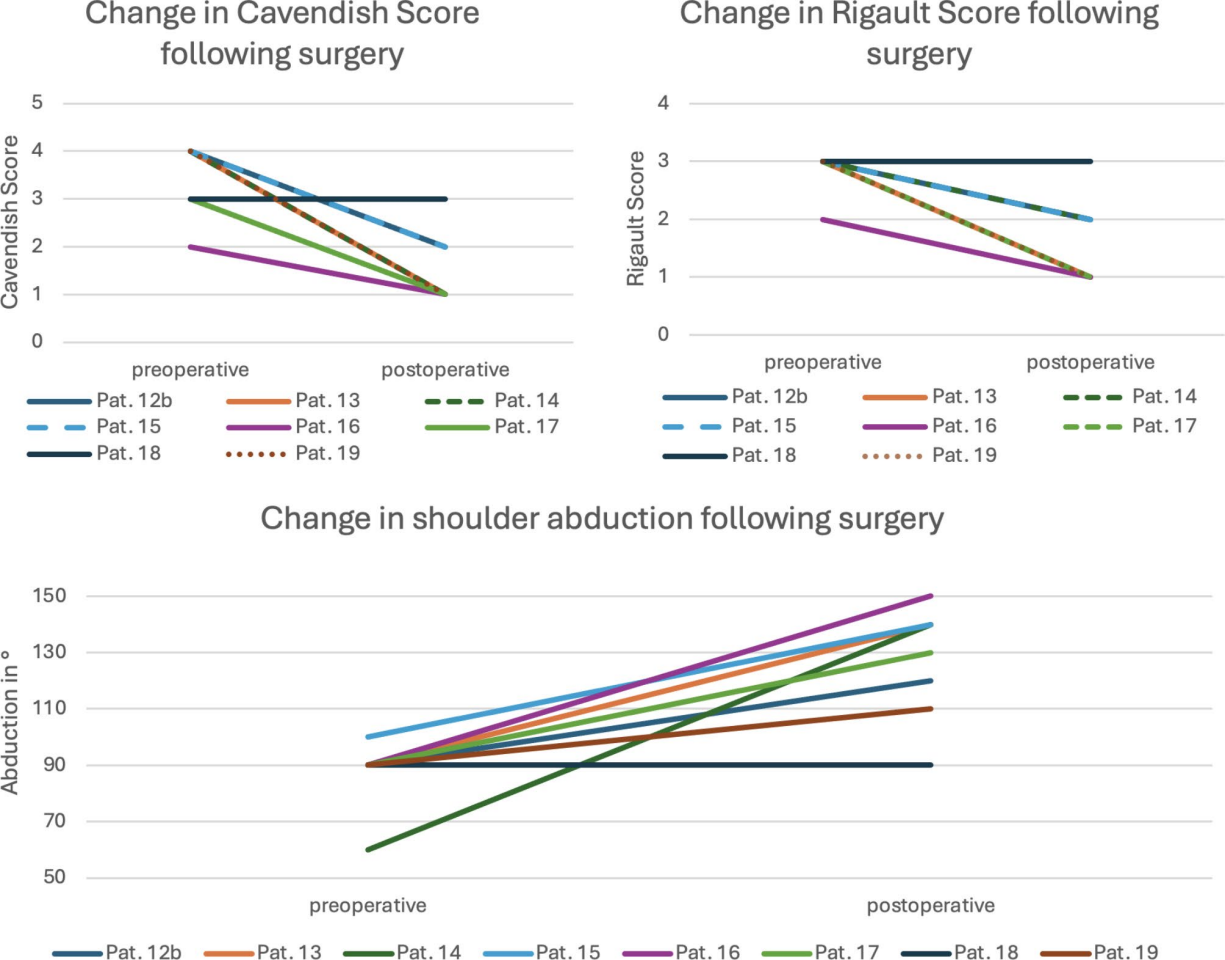
Change in Cavendish, Rigault and shoulder abduction following surgery. Included are all patients that received surgical correction of Sprengel deformity (Table 1)

**Fig. 4:**
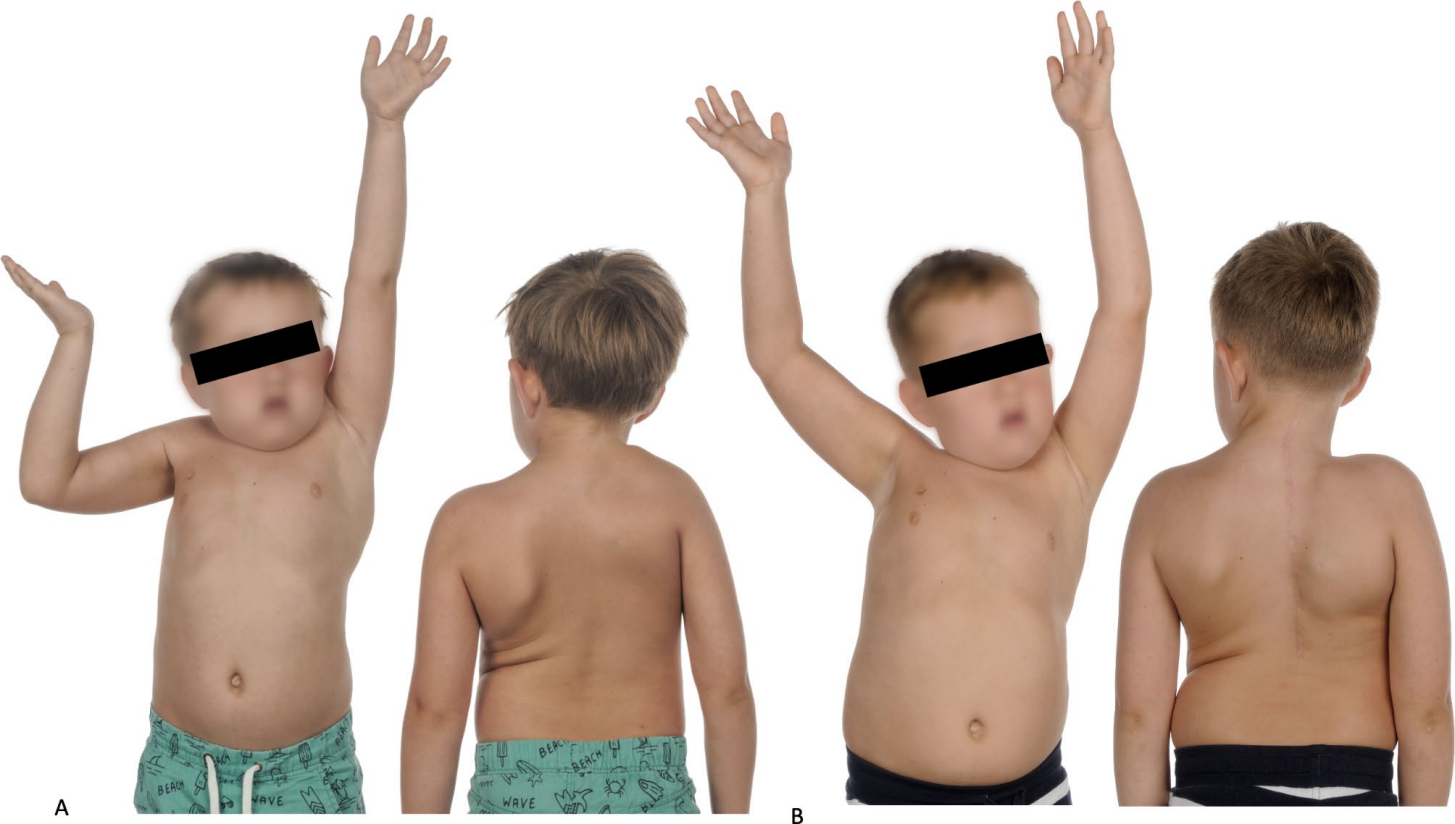
6-year-old boy with Sprengel deformity on the right side before and after reconstruction according to Green. A: Preoperative range of motion with shoulder abduction of 60°, Cavendish score 4, Rigault score 3. B: Postoperative range of motion with shoulder abduction of 140°, Cavendish score 1, Rigault score 1

Using a modified technique according to Green, the cosmetic appearance according to Cavendish was improved from a median score of 4 (IQR 3–4) to 1 (IQR 1–2) (Figs. 3 and 4).

The median time to return to daily life was 3 months (IQR 2.2–3.5).

### Radiographic outcome following surgery

The median Rigault score of the surgical cohort was lowered from 3 (IQR 3–3) to 1.5 (IQR 1–2) (Fig. 3).

### Perioperative parameters and treatment related complications

4/8 surgically treated patients showed postoperative complications. One transient radial nerve palsy and one transient brachial plexus palsy were observed and treated with physiotherapy until full recovery after five months postoperatively in each case. One pneumothorax was diagnosed intraoperatively after transcostal fixation and immediately treated by pleural drainage at the end of surgery. One seroma of the posterior chest wall was observed on the 19th postoperative day but did not need further treatment.

The median surgery time (incision-suture) was 93 min (IQR 87–96) (Table 1).

### Patient reported outcome measurement

13 of the 19 questionnaires were fully completed and used for analysis. Of those 13 patients, six patients received surgical correction and seven patients were treated conservatively. The median quickDASH score was 11.4 (IQR 7–31) in the surgical cohort and 9.1 (IQR 5–22) in the conservative cohort. The median EQ-VAS score was 81/100 (IQR 78–84/100) in the surgical cohort and 80/100 (IQR 58–95/100) in the conservative group.

## Discussion

Sprengel deformity is a rare skeletal abnormality, which leads to a limited range of motion and a visible elevation of the affected shoulder. Conservative treatment such as physiotherapy or ergotherapy can only help to establish coping mechanisms for the compensation of the limited range of motion. It cannot change the malformed structure or pathoanatomical dislocation of the scapula. Therefore, in severely affected patients (Cavendish score 3 or 4; Rigault score 3) with limited range of motion of the affected shoulder, surgery is an option to gain function and improve cosmetic appearance [8, 11].

This retrospective study was conducted to compare the clinical and radiographical outcome of patients with Sprengel deformity who either underwent conservative treatment or received surgical reconstruction using a modified Green procedure and to assess the mid-term functionality of the affected shoulder.

Recently a systemic review and meta-analysis was performed evaluating the surgical treatment of Sprengel deformity. A total of 41 case series with 674 patients and 711 affected shoulders were included, which mainly investigated the two most commonly used surgical procedures according to Woodward (234 patients) and Green (280 patients). In accordance with the results of our study surgical treatment achieved good results with an increase in shoulder range of motion of about 40° and an improvement of cosmetic appearance. Best outcomes were achieved in children younger than 8 years, even with severe deformity [22]. In our study the two oldest patients (16 years) had the least benefit with an abduction to the horizontal plane, while younger children achieved a median abduction of 130 degrees and thus being able to work above shoulder height, lift things from shelves, etc. The findings of our study thus support the recommendation to consider extensive surgery only in younger patients, ideally before the age of 8–10 years to achieve good functional outcomes.

According to the review of Zarantonello et al. the use of the Green’s procedure achieved a greater derotation of the scapula due to its change of muscle insertion at the scapula itself. The Woodward procedure on the other hand seemed to be associated with fewer complications and might be biomechanically favourable since the muscle insertions at the spine are distalised [22].

In our patients a gain of 45° in shoulder abduction through surgery was observed. This is similar with the postoperative outcome of 40° reported in literature [22]. Other studies that analysed the procedures according to Woodward and Green all seem to have similar rates in the gain of the range of motion [23, 24]. Interestingly, in the surgical cohort of our study a slight re-decrease of the initially gained shoulder abduction three months postoperatively was determined at maximum follow-up.

A great risk of these procedures is overstretching of the nerve plexus [25]. In our cohort, we had one transient plexus damage, that fully recovered within five months and a radial nerve palsy that was also completely reversible. Even though there was no permanent damage, it remains debatable if a primary osteotomy of the clavicle would have prevented these complications [8].

For adequate preoperative planning and to decrease the risk of further complications a 3D-CT scan is performed at our institution prior to the surgery to detect potential further anatomical anomalies. This recommendation is also supported by other study groups and underlines the need for meticulous preoperative assessment of the deformity [7, 14, 24].

Lately a modified surgical technique, the ‘endoscopic assisted Woodward procedure’, has been reported by Soldado et al., who performed this procedure on twelve children with Cavendish score 3 (nine patients) or 4 (three patients) [26]. With a mean improvement of the shoulder abduction of 48° these results seemed to be in concordance with our findings. Yet in the three severe cases a smaller gain of 23° was achieved [26]. The results of our study could not confirm a positive correlation between the severity of the deformity and a lesser gain of function. In our study the two oldest patients (16 years) had the least gain of shoulder range of motion.

Even though the endoscopic technique does not require a long midline incision and therefore bears the advantage of less scaring [26], the surgery-related trauma to the soft tissue probably remains the same while possibly decreasing the surgical intervention possibilities in capacity of deformity correction and prevention of intraoperative complications. In this context the knowledge of perioperative parameters such as the operation time and blood loss which were not provided by Soldado et al. [26] would be interesting for further comparison.

The timing of the surgery remains an important issue. Literature suggests the best clinical and cosmetic outcome if surgery is performed in patients under the age of eight years [22]. The age in our patient group is too heterogenous for reliable conclusions but it seems that the younger children (age less than seven years) had a greater benefit regarding function than the older children (age 16 years).

Patient reported outcome using the EQ-VAS and the quickDASH showed similar scores for patients treated conservatively and surgically, suggesting that surgery in severely affected patients can lead to a comparable functional outcome. Yet, on the other hand it supports our standard to only perform surgery on children with high functional impairment since after operative deformity correction functional restrictions usually remain to some extent.

Bae et al. and Del Corral et al. investigated the minimal clinically important difference (MCID) of the EQ-5D-Y and the EQ-VAS in pulmonal diseases and found a MCID of the EQ-VAS of 8 and 7.5 respectively [27, 28]. Likewise, Franchignoni et al. analysed the MCID of the QuickDASH and found a change of 15.9 to be meaningful and a change of 21.9 points to be of small to moderate improvement [29].

Walstra et al. analysed a cohort of six patients undergoing surgical correction in Sprengel deformity evaluating functionality of the shoulder using the Constant score, the DASH and the simple shoulder test [23]. They found a long-term DASH score of 14.6 points (range 6.6–28.3) which is comparable to the quickDASH score of our surgical cohort with 11.4 points and slightly less than in our conservatively treated patients with 9.1 points. This indicates that surgery can lead to a good functionality but cannot fully eradicate any impairments. As Sprengel deformity is most often associated with other anomalies, it is not expected to achieve a complete restitution [23].

This study has several limitations. The small cohort of patients in this case series, which is due to the rarity of the deformity and the scarcity of surgery, is a major limitation and prevents any interferential statistical analysis. If other authors would follow the same reporting standards, results could be compared in multicentre studies. Since the disease is rare, operative treatment strategy, physiotherapy and rehabilitation may change over time making it even more difficult to assess the effect of each of these elements. Ideally, multicentre studies with a predefined multidisciplinary treatment approach and prospectively collected functional outcome measures could be the next step. The treatment approach however must be defined based on the identified factors: patient age, severity of the deformity (Cavendish classification), and the patient’s and family’s willingness to undergo operative treatment.

We also inform the reader about transfer bias which can be sustained by the median follow-up of 62 months, which is longer than in pre-existing studies but in general still relatively short, and so especially changes of shoulder function may still occur. The need for long-term follow-up is underlined by our observation that initially gained abduction ability is partially lost in some patients. Assessment bias might have been caused by the fact that the patients were treated by the same authors who assessed and interpreted the data. An important point that also needs to be considered is selection bias since patients with mild deformity might not have been referred to our clinic, so that one might assume that especially patients with severe deformity and/or relevant functional impairment were included in this study.

## Conclusions

In conclusion, mild forms of Sprengel deformity with only light impairments in range of motion can be treated conservatively with physio- or ergotherapy resulting in good shoulder function. Severe deformities profit from surgical correction. The modified technique according to Green showed a satisfactory outcome in this small patient cohort. In patients with Cavendish score 3 or 4 and Rigault score 3, surgery could improve the abduction and cosmesis of the affected shoulder and resulted in a satisfactory functionality. If surgical treatment is chosen reconstruction should be conducted in younger children to achieve the best possible results, carefully taking the potential for severe complications of extensive surgery into consideration.

## Data Availability

The datasets used and/or analysed during the current study are available from the corresponding author on reasonable request.

## Abbreviations

3D-CT: Three-dimensional computer tomography
DASH: Disabilities of the Arm and Shoulder Questionnaire
IQR: Interquartile Range
MCID: Minimal Clinical Important Difference
Pat.: Patient(s)
VAS: Visual Analogue Scale
